# Partnering With Technology: Advancing Laparoscopy With Artificial Intelligence and Machine Learning

**DOI:** 10.7759/cureus.56076

**Published:** 2024-03-13

**Authors:** Taufiqa Reza, Syed Faqeer Hussain Bokhari

**Affiliations:** 1 Medicine, Avalon University School of Medicine, Youngstown, USA; 2 Surgery, King Edward Medical University, Lahore, PAK

**Keywords:** surgery, postoperative care, real-time assistance, surgical planning, risk stratification, patient selection, machine learning, artificial intelligence, minimally invasive surgery, laparoscopic surgery

## Abstract

Artificial intelligence (AI) and machine learning (ML) have emerged as transformative technologies in optimizing laparoscopic surgery, offering innovative solutions to enhance surgical precision, efficiency, and safety. This editorial explores the potential role of AI/ML across the surgical continuum, including preoperative optimization, intraoperative assistance, and postoperative care. It outlines the benefits of laparoscopic surgery compared to traditional open procedures and identifies current challenges such as technical difficulty and human error. The editorial discusses how AI and ML technologies can address these challenges, including patient selection and risk stratification, surgical planning and simulation, and personalized medicine approaches. Moreover, it examines the role of AI/ML in intraoperative assistance, such as instrument tracking and guidance, real-time tissue analysis, and the detection of potential complications. Postoperative care and follow-up are also explored, highlighting the potential of AI/ML in monitoring patient recovery, predicting and preventing complications, and tailoring rehabilitation plans. Ethical concerns surrounding data privacy and security, the lack of transparency in decision-making, potential job displacement, and regulatory frameworks are discussed as challenges to the widespread adoption of AI/ML in laparoscopic surgery. Finally, potential areas for further research and exploration are outlined, emphasizing interdisciplinary collaboration and the need for transparent and accountable AI systems. Overall, this editorial provides insights into the challenges and opportunities in harnessing AI/ML technologies to optimize laparoscopic surgery and improve patient outcomes.

## Editorial

Introduction

Laparoscopic surgery, also known as minimally invasive surgery, is a revolutionary technique that has transformed countless surgical procedures. Unlike traditional open surgery, which requires large incisions, laparoscopy utilizes small keyhole incisions and specialized instruments for operating inside the abdomen or pelvis. This approach offers several advantages over traditional open surgery, including reduced postoperative pain, shorter hospital stays, faster recovery times, and improved cosmetic outcomes. The minimally invasive nature of laparoscopic surgery also leads to decreased blood loss and lower rates of complications such as infection and scarring [1,2]. However, despite its numerous benefits, laparoscopic surgery presents certain challenges that can impact patient outcomes. One of the primary challenges is the technical difficulty associated with manipulating instruments in a confined space while viewing a two-dimensional video feed. Surgeons must undergo extensive training to develop the necessary hand-eye coordination and spatial awareness required to perform laparoscopic procedures effectively. Additionally, human error remains a concern, as even experienced surgeons may encounter difficulties in accurately assessing tissue characteristics or navigating complex anatomical structures.

In recent years, there has been a growing interest in leveraging artificial intelligence (AI) and machine learning (ML) technologies to address the challenges inherent in laparoscopic surgery. AI refers to the simulation of human intelligence by computer systems, enabling them to perform tasks that typically require human cognition, such as problem-solving and decision-making. ML, a subset of AI, involves the development of algorithms that allow computers to learn from data and improve their performance over time without explicit programming. The emergence of AI and ML in the field of laparoscopic surgery holds promise for enhancing surgical precision, efficiency, and safety. These technologies have the potential to augment the capabilities of surgeons by providing real-time assistance and decision support during procedures. For example, AI algorithms can analyze live video feeds from laparoscopic cameras to identify anatomical landmarks, highlight critical structures, and track the movement of surgical instruments. By integrating computer vision techniques with ML algorithms, AI systems can assist surgeons in achieving optimal positioning and manipulation of instruments, thereby reducing the risk of errors and complications. Furthermore, AI-powered predictive analytics tools can analyze patient data, such as medical imaging scans and electronic health records, to provide personalized insights and recommendations for treatment planning and surgical approach selection. By analyzing large datasets of past surgical cases and outcomes, ML algorithms can identify patterns and trends that may inform clinical decision-making and improve patient outcomes.

Preoperative optimization

Preoperative optimization plays a crucial role in ensuring favorable surgical outcomes by identifying patient-specific factors, assessing risks, and planning appropriate interventions. AI and ML offer innovative approaches to enhance preoperative optimization processes, providing valuable insights and personalized recommendations tailored to individual patient needs. Patient selection and risk stratification represent fundamental aspects of preoperative optimization, aiming to identify patients who are most likely to benefit from surgery while minimizing potential risks and complications. Traditionally, patient selection relies heavily on surgeon experience and judgment. AI/ML algorithms can analyze vast databases of patient data, including medical history, laboratory results, and imaging studies, to stratify patients based on their risk profiles. By identifying factors associated with adverse outcomes, such as comorbidities, physiological parameters, and procedural complexity, these algorithms can assist clinicians in making informed decisions regarding patient eligibility for surgery and optimizing perioperative care strategies. Identifying high-risk patients allows for targeted pre-habilitation and the implementation of personalized pre-surgical interventions, such as targeted exercises or nutritional adjustments, to optimize their health and improve surgical outcomes.

Laparoscopic surgery requires meticulous preoperative planning to ensure efficient and safe execution. AI-driven surgical planning platforms leverage advanced imaging techniques, such as MRI and CT scans, to generate three-dimensional reconstructions of patient anatomy. ML algorithms can then analyze these reconstructions to identify anatomical variations, predict surgical challenges, and simulate different surgical approaches. By integrating biomechanical models and surgical databases, AI-powered simulation tools enable surgeons to practice procedures virtually, refine surgical techniques, and anticipate potential complications, ultimately enhancing surgical precision and patient safety.

Personalized medicine approaches aim to tailor treatment strategies to the individual characteristics and preferences of each patient, optimizing therapeutic outcomes while minimizing risks and adverse effects. AI and ML algorithms play a pivotal role in personalized medicine by analyzing multidimensional datasets, including genomic profiles, biomarker expression patterns, and treatment response data. By applying predictive analytics and pattern recognition techniques, these algorithms can identify patient-specific factors that influence treatment response and prognosis, guiding clinicians in selecting the most effective interventions and optimizing treatment regimens. Furthermore, AI-driven decision support systems can integrate patient-specific data with evidence-based guidelines, clinical protocols, and expert knowledge to generate personalized treatment recommendations. These systems leverage natural language processing and semantic analysis techniques to extract relevant information from electronic health records, medical literature, and clinical practice guidelines, providing clinicians with real-time guidance and decision support at the point of care.

Intraoperative assistance

The intraoperative phase of laparoscopic surgery is a critical juncture demanding precise manipulation and real-time decision-making. AI and ML offer innovative solutions to assist surgeons in overcoming these challenges and optimizing intraoperative outcomes. By providing real-time assistance and decision support, AI/ML systems enhance surgical accuracy, streamline workflow, and improve patient care. Instrument tracking and guidance represent essential aspects of intraoperative assistance, enabling surgeons to navigate complex anatomical structures and manipulate surgical instruments with precision. AI/ML algorithms can analyze live video feeds from laparoscopic cameras or robotic surgical systems to track the movement and position of instruments in real time. By employing computer vision techniques and pattern recognition algorithms, these systems can accurately identify and track the location of surgical instruments relative to anatomical landmarks, facilitating precise instrument manipulation and minimizing the risk of inadvertent tissue damage. Furthermore, AI-driven instrument tracking systems can provide surgeons with augmented reality overlays or visual cues superimposed onto the surgical field, guiding them to target locations and optimal trajectories for instrument insertion. By integrating preoperative imaging data and intraoperative navigation technologies, AI/ML-guided surgical systems enable surgeons to visualize the internal anatomy in three dimensions and plan their approach with enhanced spatial awareness and precision. Real-time tissue analysis and identification are critical for ensuring optimal surgical outcomes and minimizing the risk of inadvertent tissue injury or damage. AI/ML algorithms can analyze live video feeds or intraoperative imaging data to perform real-time tissue classification and characterization. By leveraging advanced image processing techniques and ML models, these systems can differentiate between different types of tissue (e.g., healthy tissue, tumors, and blood vessels) based on their visual appearance and texture features.

The detection of potential complications during surgery is essential for timely intervention and mitigating adverse outcomes. ML algorithms can continuously analyze video and sensor data from the operating room to detect potential bleeding, unexpected tissue changes, or other anomalies that might indicate an impending complication. Early detection allows for immediate intervention, potentially mitigating the severity of complications and improving patient outcomes. AI models trained on historical data can analyze real-time patient data (e.g., vital signs and blood gas levels) during surgery to predict the risk of potential complications. This information can help surgeons anticipate challenges and take preventative measures, ensuring patient safety throughout the procedure. By offering real-time guidance, tissue analysis, and complication detection, AI and ML act as valuable adjuncts to surgeons in the operating room. This collaborative approach holds immense potential for enhancing surgical precision, minimizing errors, and ultimately leading to improved patient outcomes and safety during laparoscopic surgery.

Postoperative care and follow-up

Postoperative care and follow-up play vital roles in patient recovery post surgery, encompassing monitoring, complication management, and long-term outcome optimization. AI and ML offer promising avenues for improving postoperative care through real-time monitoring, predictive analytics, and personalized interventions tailored to individual patients [3]. AI/ML algorithms can continuously analyze physiological data to monitor patient progress and detect early signs of complications, aiding in timely intervention. Remote monitoring technologies equipped with AI capabilities extend this surveillance beyond hospital settings, enhancing early complication detection. Additionally, predictive models developed from large datasets enable risk assessment and personalized complication prevention strategies, reducing morbidity and mortality. AI-driven decision support systems provide real-time recommendations based on patient-specific factors, optimizing perioperative care and mitigating complications. Moreover, AI/ML technologies facilitate tailored rehabilitation plans by analyzing patient data and adjusting interventions based on real-time feedback. This personalized approach maximizes functional recovery and patient independence post surgery.

Challenges and future directions

Challenges and future directions in AI and ML in laparoscopic surgery encompass a range of considerations, including ethical concerns, transparency issues, workforce implications, and regulatory frameworks [4]. One prominent challenge revolves around the ethical implications of data privacy and security in the context of AI- and ML-driven surgical technologies. Ensuring the confidentiality and integrity of patient data is paramount, requiring robust safeguards to protect sensitive health information from unauthorized access or misuse. Additionally, the lack of transparency and explainability in AI/ML decision-making poses a significant challenge, as complex algorithms may produce outcomes that are difficult to interpret or justify. Surgeons and healthcare providers must grapple with the need for transparent and accountable AI systems that can explain their reasoning and decision-making processes in a manner that is understandable and accessible to users.

Moreover, the potential for job displacement in the surgical field raises concerns about the impact of AI and automation on the healthcare workforce. While AI-powered surgical technologies have the potential to enhance surgical efficiency and outcomes, there is a need to address workforce implications and ensure that healthcare professionals receive adequate training and support to adapt to technological advancements. Furthermore, the development and deployment of AI-powered surgical tools necessitate robust regulatory and legal frameworks to ensure patient safety, efficacy, and accountability. Regulatory agencies must establish clear guidelines and standards for the validation, approval, and oversight of AI-driven medical devices while also addressing the liability and ethical considerations associated with their use in clinical practice.

Despite these challenges, there are numerous opportunities for further research and exploration in the field of AI and ML in laparoscopic surgery. Potential areas for future research include the development of advanced computer vision algorithms for real-time image analysis and intraoperative guidance, the integration of AI-driven predictive analytics for personalized treatment planning and risk stratification, and the implementation of autonomous robotic systems for surgical assistance and teleoperation. Additionally, interdisciplinary collaboration between clinicians, engineers, data scientists, and ethicists is essential to address the multifaceted challenges and opportunities associated with AI and ML in laparoscopic surgery and pave the way for the future of surgical innovation and patient care.
